# The Distributions and Boundary of Two Distinct, Local Forms of Japanese Pond Frog, *Pelophylax porosus brevipodus*, Inferred From Sequences of Mitochondrial DNA

**DOI:** 10.3389/fgene.2018.00079

**Published:** 2018-04-05

**Authors:** Yukari Nagai, Toshio Doi, Kunio Ito, Yoshiaki Yuasa, Takeshi Fujitani, Jun-ichi Naito, Mitsuaki Ogata, Ikuo Miura

**Affiliations:** ^1^Department of Biology, Graduate School of Science, Hiroshima University, Higashihiroshima, Japan; ^2^Environmental Assessment and Symbiosis Promotion Division, Kobe Municipal Office, Kobe, Japan; ^3^Kawasaki Senior High School Attached to Kawasaki Medical School, Kurashiki, Japan; ^4^Himeji City Aquarium, Himeji, Japan; ^5^Higashiyama Zoo and Botanical Gardens Information, Nagoya, Japan; ^6^Society for the Study of Natural History of Nishi-Chugoku Mountains, Hiroshima, Japan; ^7^Preservation and Research Center, The City of Yokohama, Yokohama, Japan; ^8^Amphibian Research Center, Hiroshima University, Higashihiroshima, Japan

**Keywords:** Japanese pond frog, *cytochrome b*, D-loop, *SOX3*, two major forms

## Abstract

The Nagoya Daruma pond frog *Pelophylax porosus brevipodus* is distributed in western Japan and is traditionally divided into two local forms: the Okayama form in the west and the Nagoya form in the east. These two forms are genetically differentiated, but have never been defined taxonomically because their distributions are unclear to date. To complete the distributions and identify the boundary of the two forms, we genetically investigated 16 populations including eight populations located within the unexamined area. We found that the distributional boundary is located within a small area of Hyogo Prefecture where haplotypes of mitochondrial *cytochrome b* (*cytb*) and D-loop region corresponding to the two forms co-existed. On the other hand, the polymorphic site of the nuclear gene *SOX3* revealed introgression over the boundary into Okayama *cytb* clade. These results suggest that the two forms were geographically isolated from each other in the past, and secondarily contacted and then accepted one-way introgression. As a next step of the research, taxonomic approach is expected to define the two forms.

## Introduction

**FIGURE 1:**
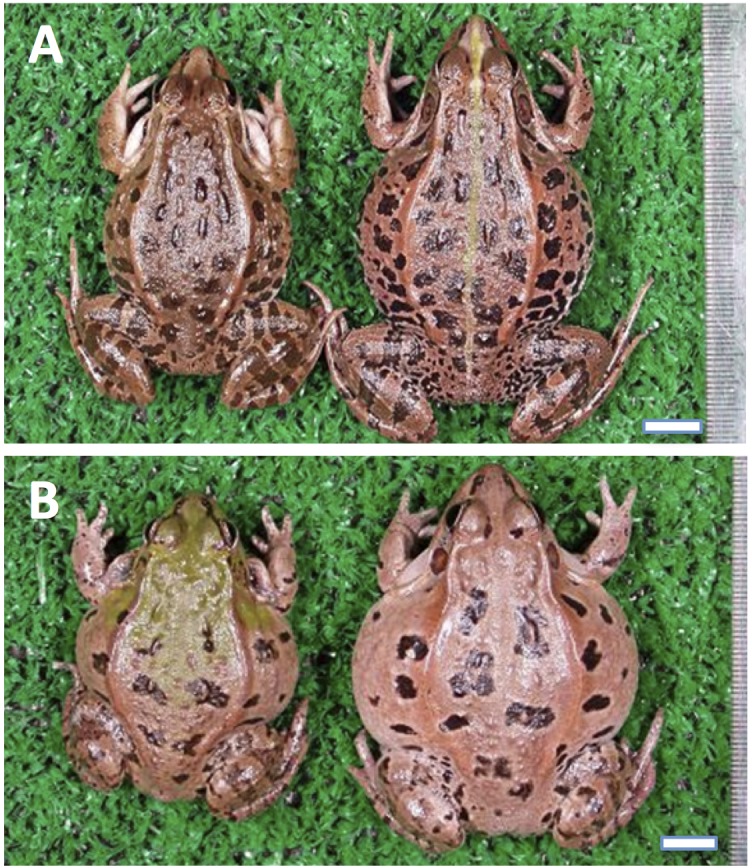
External appearance of *Pelophylax porosus brevipodus* belonging to the two local forms. **(A)** The Nagoya form and **(B)** the Okayama form. Males and females are placed on the left and right, respectively. Central line on the back is seen in the female of Nagoya form. The line is absent in all frogs of Okayama form ever examined. The black spots on the back are larger and lower in number in the Okayama form than Nagoya form.

Two pond frog species live in the Japanese islands, *Pelophylax nigromaculatus* and *Pelophylax porosus*. The latter species is endemic to Japan and is called the Daruma pond frog. It is similar to a traditional Japanese Daruma doll with its round shape. This species is comprised of two subspecies: *P. p. porosus* (Tokyo Daruma pond frog), which is distributed in eastern Japan, and *P. p. brevipodus* (Nagoya Daruma pond frog), which is distributed in western Japan. *P. p. brevipodus* is traditionally divided into two distinct, local forms called the Okayama form in the west and the Nagoya form in the east (Ito, 1941; Moriya, 1954; Kawamura, 1962; Matsui and Hikida, 1985). They are genetically differentiated from each other by their external morphologies (**Figure 1**), mating calls, sex chromosomes, allozymes and mitochondrial genes (Moriya, 1951, 1954; Nishioka et al., 1992; Nishioka and Sumida, 1994; Ueda, 1994; Sumida et al., 1998, 2000a,b; Komaki et al., 2015). However, the two forms have never been defined taxonomically because their distributions are unclear. Since the genetic researches on the two local forms to date were always restricted to several representative populations, the area covering around 150 km between the two forms remains unstudied. It is still unknown whether the two forms are geographically separated or distributed sympatrically with mutual genetic introgression. Such information is definitely necessary for judging taxonomic positions of the two forms. Recently, the geographic populations of the Okayama form have been declining and are concerned about their possible extinction (Okochi et al., 1997). The degradation is especially severe in the western edge of the distribution, Hiroshima Prefecture, where only a few tiny populations have survived (Naito et al., 2014). Conservation of the population and environment is an urgent issue and taxonomic definition of the form is expected to assist the conservation activities.

**FIGURE 2:**
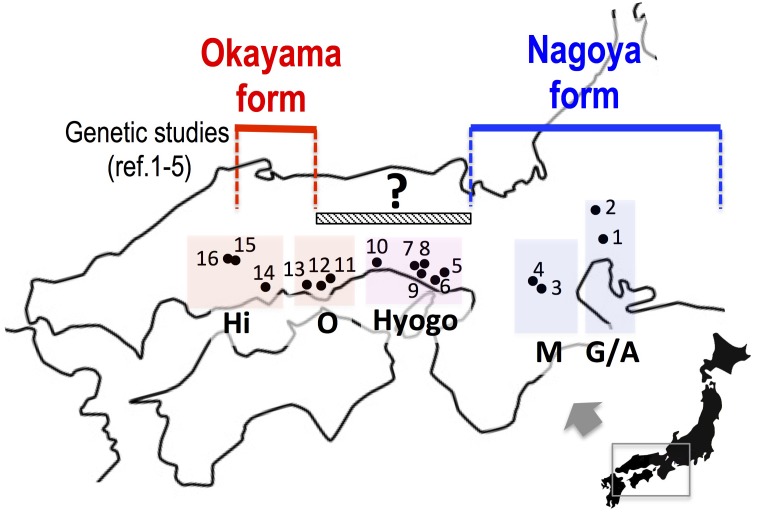
The collecting localities of *Pelophylax porosus brevipodus.* Populations 5 to 12, which are placed under a hatched bar (150 km) with a question mark, have never been examined genetically since the primary morphological study of Moriya (1954). References 1–5: Nishioka et al. (1992), Sumida et al. (1998, 2000a,b), Komaki et al. (2015). See the localities (shown in number) in **Table 1**. Hi, Hiroshima prefecture; O, Okayama prefecture; Hyogo, Hyogo prefecture; M, Mie prefecture; G/A, Gifu and Aichi prefectures.

In this study, we collected samples of the two major forms in western Japan and investigated sequences of mitochondrial and nuclear genes in order to assess whether the two forms are separated geographically or are distributed sympatrically with mutual genetic introgression. In particular, the eight populations in Okayama and Hyogo Prefectures are located between the known distributions of the two forms and were genetically examined for the first time in 63 years since the primary morphological study of Moriya (1954) (**Figure 2**).

**Table 1:** Populations, haplotype of mitochondrial *cytochrome b*, D-loop region, and genotype of nuclear *SOX3*.

Locality No.	Species	Population	Prefecture	City	Town or area	No. of frogs (♂, ♀, juvenile)	*cytb* haplotype	Repeats in D-loop region	*SOX3* (233rd)
1	*Pelophylax*	K-Nagoya	Aichi	Kita-Nagoya	Shikatsu	3 (2,1,0)	A1, A3	AB	GG
		
2	*porosus*	Gifu	Gifu	Gifu		3 (1,2,0)	A2, A3, A4		GG
3	*brevipodus*	Iga A	Mie	Iga	(A)	4 (1,3,0)	B1		GG
4		Iga B		Iga	(B)	2 (0,2,0)	B3	ABAB	GG
		
5		Kobe-O	Hyogo	Kobe	Oshibedani	9 (7,2,0)	B1	AB	GG
6		Kobe-H		Kobe	Hirano-machi	10 (3,2,5)	B1		GG
7		Kakogawa-YA		Kakogawa	Yahata (A)	30 (3,6,21)	B2, B1,C3		GT, TT
8		Kakogawa-YB		Kakogawa	Yahata (B)	6 (1,1,4)	B1, C3		GG, GT, TT
9		Kagogawa-I		Kakogawa	Inami	10 (2,5,3)	B1, C3	ABABAB, ABA	GT, TT
10		Ako		Ako	Fukuura	12 (4,8,0)	C2, C3		GT, TT
		
11		Okayama-S	Okayama	Okayama	Seto	11 (5,5,1)	C1, C3	ABA	TT
12		Okayama-N		Okayama	Nodono	20 (5,11,4)	C1		GG, GT, TT
13		Kurashiki		Kurashiki	Mabi	8 (1,2,5)	C1		TT
		
14		Fukuyama	Hiroshima	Fukuyama	Kannabe	8 (3,5,0)	C1	ABA	TT
15		Miyoshi-Y		Miyoshi	kisa, Yasuda	10 (0,0,10)	C1	ABA	TT
16		Miyoshi-K		Miyoshi	Kisa, Kaitahara	10 (0,0,10)	C1	ABA	TT

	*P. p. porosus*	Itako	Ibaraki	Itako		11 (5,6,0)	P1,P2,P3,P4		GG
	*P. nigromaculatus*	Outgroup	Hiroshima	Miyoshi	Kisa, Kaitahara	1 (0,0,1)			GG
	*P. fukienensis*	Outgroup	Taiwan				AB029941.1		

## Materials and Methods

### Frogs

The number of frogs of *Pelophylax porosus brevipodus*, *P. p. porosus* and *P. nigromaculatus* used for sequence analyses are listed in **Table 1** and their collecting locations are shown in **Figure 2**. We collected three frogs of *P. p. brevipodus* each from Aichi and Gifu Prefectures, and reared them at our laboratory, while all other tissue samples were taken from the toe-clips in the fields, and stored in 100% ethanol until use. The frogs were thereafter released to the fields. Animal care and experimental procedures were conducted under approval of the Committee for Ethics in Animal Experimentation at Hiroshima University (Permit Number: G13-3).

### DNA Extraction and PCR Amplification

Genomic DNA was extracted from the tissue samples using DNeasy blood and tissue kit (QIAGEN) according to the manufacture’s instruction. Mitochondrial *cytochrome b* and nuclear *SOX3* fragments were amplified in 50 μl solution including 1.0 μl of DNA solution, 0.2 μl GXL Taq polymerase (TaKaRa), 5 μl of 10× Buffer, 4 μl of 2.5 mM dNTP, and 1 μl of 12.5 mM primers at 98°C for 5 s followed by 30 cycles of 98°C 10 s, 64°C for 40 s, and 72°C for 60 s. The amplified product was purified using GFX PCR DNA and Gel band purification kit (GE Healthcare), and was used for nucleotide sequence determination with 3130XL sequencing machine (ABI).

Mitochondrial fragments including D-loop region (300∼500 bp) were amplified and purified by the above methods, and were cloned into pUC118 vector using Mighty cloning kit (TaKaRa) with competent cell DH5α (Ecos, Nippon gene) according to the manufactures’ instructions. One to three colonies were picked up and the nucleotide sequences were determined by the method described above. Gene trees were constructed based on the nucleotide sequence of cytochrome-b gene by the methods of maximum likelihood (ML), neighbor joining (NJ) and maximum parsimony (MP) methods using Mega 7 software (Kumar et al., 2016). *p*-distance was also calculated using the above software. Primers used are forward 5′-CCA TGC ACT ACA CAG CCG ACA-3′ and reverse 5′-AGG TTT TTG CGA TAG GGC GGA-A3′ for *cytochrome b* (designed in this study using software Genetix ver. 7.3, Genetix corp.), S1 5′-GTG CGC TCC TCC TGC TTC TTT-3′ and A1 5′-TCC TCA AGT TTT CTG CAT TCT GAT-3′ for *SOX3* (Miura et al., 2016), and F23 5′-ATG AAT GCT ATA ATG ACA TAA TGT-3′ and R21 5′-TGC TGG CTC CTA AGG CCA GTG GAG GGC TGT-3′ for D-loop region (Sumida et al., 2000a). The sequences of ten haplotypes (A1–4, B1–3, and C1–3) of *cytochrome b* have been deposited with the DDBJ Data Libraries under the accession numbers LC217488-LC217457, and the sequences of *SOX3* (Kurashiki and Iga populations), under the accession numbers LC316654 and LC316655, respectively.

## Results

### Mitochondrial *Cytochrome b*

**FIGURE 3:**
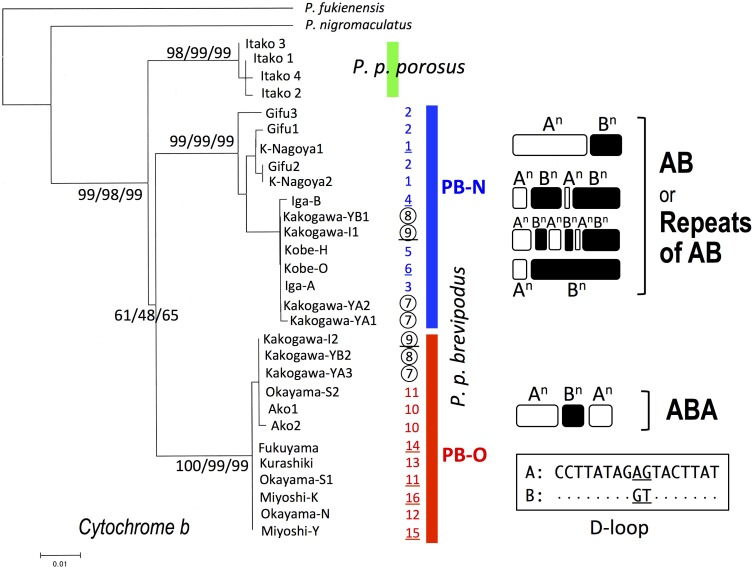
Maximum-likelihood (ML) tree of *Pelophylax porosus brevipodus* collected from 16 populations based on mitochondrial cytochrome-b gene and the repeating pattern of 17-bp sequences in D-loop region. The numbers at each node on the tree are ML/NJ/MP bootstrap values. The numbers put alongside vertical bars are population numbers shown in **Table 1** and **Figure 2**: the Kakogawa populations 7, 8, and 9 are circled and the populations, of which D-loop regions were examined, are underlined. Repeats of type A and B in D-loop region are indicated by open and closed round squares, respectively, and the sequences of type A and B are shown in the box. Dots in type B indicate the same nucleotides as type A.

**FIGURE 4:**
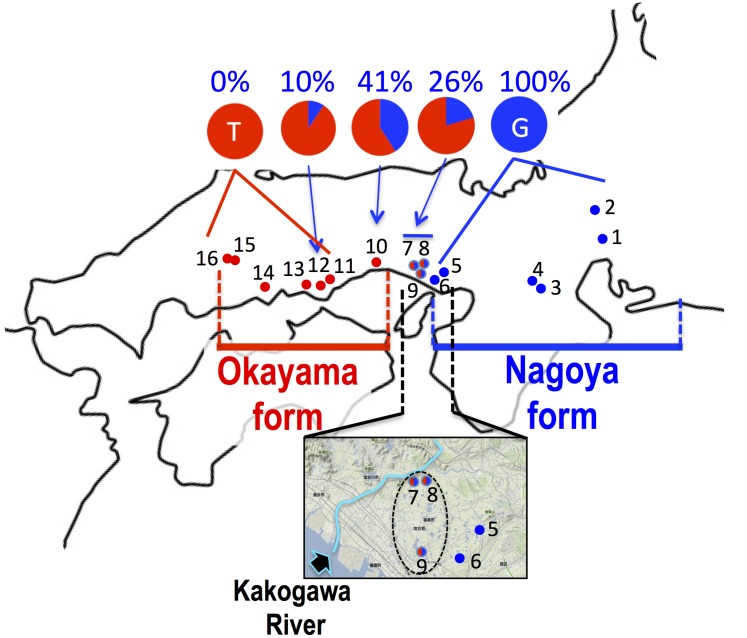
Haplogroup of *cytochrome b* and frequency of haplotype at 233rd position of *SOX3* in 16 populations of *P. p. brevipodus*. The red and blue circles indicate haplogroups of clade PB-O and PB-N, respectively. Red-blue (half-half) circles indicate sympatric distribution of the two haplogroups within a single population. The area including populations 5–9 are magnified with an arrow showing Kakogawa River. The frequencies of thymine and guanine haplotypes at 233rd positions of *SOX3* are indicated in red and blue, respectively. See the localities (shown in number) in **Table 1**.

We collected samples consisting of 156 specimens (38 males, 55 females, and 63 juveniles) from 16 populations covering their present habitat in western Japan (**Figure 2** and **Table 1**). We determined the nucleotide sequences of 566 base pairs of the mitochondrial cytochrome-b gene. Ten haplotypes were identified and the gene tree was constructed using the maximum likelihood (ML) method (**Figure 3** and **Table 1**). The haplotypes formed two distinct clades, which are designated PB-N and PB-O because they correspond to the Nagoya form and Okayama form, respectively. The genetic (*p*) distance between the two clades was 0.055 and that between the two subspecies was 0.054, suggesting that the genetic relationships among *P. p. porosus* and the two local forms of *P. p. brevipodus* are within almost equal range of each other. Notably, two haplotypes of PB-O and PB-N were detected in Kakogawa-YA, -YB and -I populations of Hyogo Prefecture (7, 8, and 9 in **Figures 2**–**4**), which were located immediately east over the Kakogawa River. Three of 30, one of six, and three of ten specimens examined in the populations had PB-O haplotypes, while the others possessed PB-N haplotypes. This indicates that the boundary between the two clades is restricted to the small area of Hyogo Prefecture.

### Repeated Sequence in D-loop Region

The D-loop region of the mitochondrial genome includes a highly repeated sequence. We cloned this region and determined the nucleotide sequences of specimens from eight populations of *P. p. brevipodus* (**Table 1**). The repeated region comprised of repeats of two kinds of 17-bp units designated types A and B of which nucleotides at the 9th and 10th positions were different: AG and GT, respectively (**Figure 3** and Supplementary Table 1). The repeated pattern was different among populations (**Figure 3** and Supplementary Table 1). Pattern AB was specific to the Kita-Nagoya population (population No. 1 in **Table 1** and **Figure 3**), while pattern ABA was observed in the Kakogawa I2, Okayama-S1 and three Hiroshima populations (9, 11, and 14–16). In the Iga, Kobe-O and Kakogawa I1 populations (4, 6, and 9), the observed patterns were ABAB, AB, and ABABAB, respectively. All the repeat patterns were thus classified into two types: AB (or repeats of AB) and ABA. The two types corresponded with the two major clades of *cytochrome b*: PB-N with AB or repeats of AB and PB-O with ABA, respectively. In the Kakogawa-I population (9), the specimen with the PB-N *cyt-b* haplotype had the ABABAB pattern while that with the PB-O haplotype possessed the ABA pattern.

### *SOX*3

The sequence of 860 base pairs of the nuclear *SOX3* gene was determined for 140 specimens from 16 populations. The nucleotide at position 233 varied by population (**Figure 4** and **Table 1**). In the six eastern populations (1–6: Kita-Nagoya, Gifu, Iga-A, Iga-B, Kobe-O, and Kobe-H), all specimens were homozygous for guanine. On the other hand, in the five western populations (11, 13–16: Okayama, Kurashiki, and three of Hiroshima Prefecture), all were homozygous for thymine. In the other five populations (7–10, 12) located at the intermediate regions, the specimens were heterozygous or homozygous for guanine or thymine (**Figure 4**). The frequency of guanine in these populations varied from 10 to 41%.

## Discussion

Based on the mitochondrial *cytochrome b*, the two major *P. p. brevipodus* forms of Okayama and Nagoya were identified as distinct clades, and two major types of the D-loop region supported the *cytb* clades. The genetic distance (*p*-distance, 0.055) between the two forms was very similar to that (0.054) between the two subspecies. These genetic relationships are well supported by another study that used mitochondrial and nuclear genes (Komaki et al., 2015). The distribution boundary between the two forms was for the first time found in this study. It is located at a very small area that included the Kakogawa populations (7–9 in **Figures 2**, **4**) of Hyogo Prefecture and was where two haplotypes of the Okayama and Nagoya forms co-existed. This shows that the two forms were geographically isolated from each other in the past and have secondarily contacted at the small area after they were genetically differentiated. The molecular clock based on *cytochrome b* and seven nuclear genes estimates that the two forms were separated from each other around 1.3 MYA (Komaki et al., 2015). Currently, no remarkable barrier of geographic structure could be identified around the boundary that separates the two forms of *Pelophylax porosus brevipodus*, or no geographic event that actually occurred 1.3 MYA is known. However, some geographic barrier must have existed in the past and prevented crossings across the boundary area, because many other animals, such as grasshopper, harvestman, frog, landing snail, and monkey, are likewise genetically differentiated between the west and the east of the boundary region (Tsurusaki et al., 1991; Kawakami, 1999; Nishi and Sota, 2005; Kawamoto et al., 2007; Nishizawa et al., 2011). Conversely, it was found that nuclear gene *SOX3* showed introgression over the boundary from eastern Nagoya form into the western Okayama form. The genetic affinity between the two forms is also confirmed by the results of artificial crossings in the study of Moriya (1960a,b), showing fertile hybrids between the two forms. However, it was quite difficult in this study to recognize the genetic introgression in external morphology: for example, a central line on the back, which was normally observed in 44% frogs of Nagoya form, was not found in any populations of the Okayama form (except one specimen in Kakogawa I population, No. 9 on the map). A deeper analysis on nuclear genomes of the two forms focusing on the populations around the boundary is required to verify the on-going introgression of the genomes.

## Conclusion

It is evident that the two local forms had been once isolated from each other and accumulated their genetic differences, and thereafter they have secondarily contacted immediately east over the Kakogawa River (**Figure 4**) and possibly the Okayama form is now accepting introgression from the Nagoya form. We speculate that the ancestral lineage of the Okayama form remains around the eastern edge of the range (Hiroshima Prefecture and the western region of Okayama Prefecture in **Figure 2**) where population declining and extinction are concerned. At a next step of the research, taxonomic definition of the two forms are expected (for example, name of Okayama Daruma pond frog is given to the Okayama form), because they are precisely identified and the geographic boundary between the two forms is very clear based on the mitochondrial DNA. Unfortunately, the previous study on morphology (Moriya, 1954) used no statistical analyses and examined only one population of the Okayama form, and the previous mating call analysis (Ueda, 1994) was restricted to just one or two populations of each form, which are located at the extremes in distribution. Hence, a future taxonomic approach needs to consider the distribution range for choosing populations and complete investigation on the morphology and mating calls of the two forms.

## Author Contributions

IM and MO designed the study and wrote the manuscript. YN, MO, and IM performed the experiments and analyses. TD, KI, YY, TF, and J-iN collected the specimens and discussed about the results. All authors read and approved the final manuscripts.

## Conflict of Interest Statement

The authors declare that the research was conducted in the absence of any commercial or financial relationships that could be construed as a potential conflict of interest. The handling Editor declared a past co-authorship with the authors MO and IM.
